# *β*-Sitosterol Glucoside-Loaded Nanosystem Ameliorates Insulin Resistance and Oxidative Stress in Streptozotocin-Induced Diabetic Rats

**DOI:** 10.3390/antiox11051023

**Published:** 2022-05-22

**Authors:** Sherif M. Afifi, Naglaa M. Ammar, Rabab Kamel, Tuba Esatbeyoglu, Heba A. Hassan

**Affiliations:** 1 Pharmacognosy Department, Faculty of Pharmacy, University of Sadat City, Sadat City 32897, Egypt; 2 Therapeutic Chemistry Department, National Research Centre, 33 El Bohouth St., Dokki, Giza 12622, Egypt; nm.ammar@nrc.sci.eg (N.M.A.); ha.el-saud@nrc.sci.eg (H.A.H.); 3 Pharmaceutical Technology Department, National Research Centre, Cairo 12622, Egypt; rk.mahmoud@nrc.sci.eg; 4 Department of Food Development and Food Quality, Institute of Food Science and Human Nutrition, Gottfried Wilhelm Leibniz University Hannover, Am Kleinen Felde 30, 30167 Hannover, Germany

**Keywords:** antidiabetic, glucagon, insulin resistance, malondialdehyde, nanoemulsion, oxidative stress, release study, SEDDS, *Senecio petasitis*

## Abstract

*β*-Sitosterol glucoside (SG), isolated from *Senecio petasitis* (Family Asteraceae), was loaded in self-nanoemulsifying drug delivery systems (SEDDS) in a trial to enhance its solubility and biological effect. Various co-surfactants were tested to prepare a successful SEDDS. The selected SG-loaded SEDDS had a droplet size of 134 ± 15.2 nm with a homogenous distribution (polydispersity index 0.296 ± 0.02). It also demonstrated a significant augmentation of SG in vitro release by 4-fold compared to the free drug suspension. The in vivo insulin sensitivity and antidiabetic effect of the prepared SG-loaded SEDDS were further assessed in streptozotocin-induced hyperglycemic rats. The hypoglycemic effect of SG-loaded nanosystem was evidenced by decreased serum glucose and insulin by 63.22% and 53.11%, respectively. Homeostasis model assessment-insulin resistance (HOMA-IR) index demonstrated a significant reduction by 5.4-fold in the diabetic group treated by SG-loaded nanosystem and exhibited reduced glucagon level by 40.85%. In addition, treatment with SG-loaded nanosystem significantly decreased serum MDA (malondialdehyde) and increased catalase levels by 38.31% and 64.45%, respectively. Histopathological investigations also supported the protective effect of SG-loaded nanosystem on the pancreas. The promising ability of SG-loaded nanosystem to ameliorate insulin resistance, protect against oxidative stress, and restore pancreatic *β*-cell secretory function warrants its inclusion in further studies during diabetes progression.

## 1. Introduction

Diabetes mellitus (DM) is a metabolic disease marked by excessively high blood glucose levels because of insufficient endogenous insulin production or activity. As a result of increased intake of high-calorie meals and greater adoption of a sedentary lifestyle, its incidence has increased. It is a serious public health issue around the world. In 2017, more than 425 million people were diagnosed with diabetes [1]. Microvascular complications, such as nerve injury (neuropathy), renal system damage (nephropathy), eye damage (retinopathy), cardiovascular disease, stroke, and peripheral vascular disease, are all indications of diabetes complications [2]. Type 2 diabetes mellitus (T2DM) can cause long-term production of reactive oxygen species and damage to organs like the eyes, nerves, kidneys, and blood vessels if not treated properly [3]. Diet therapy, pharmacology, and insulin therapy are the basic treatment approaches for diabetes control, along with a large range of glucose-lowering medications that exert hypoglycemic effects through diverse mechanisms [4]. These therapy alternatives, however, have not gained much traction because they are frequently linked with limitations such as medication resistance, side effects, and toxicity. Due to the safe and non-toxic aspect of herbal-based medications, supplementation of hypoglycemia pharmaceutical drugs to treat DM is now a potential and unique therapeutic method, regardless of the presence of these hypoglycemic pharmacological drugs [5]. Insulin resistance is a condition in blunted response to insulin stimulation of target tissues [6], and it manifests as hyperglycemia and compensative hyper-insulinemia [7].

*Senecio petasitis*, also known as *Roldana petasitis* or velvet groundsel, is an evergreen shrub of the family Asteraceae/Compositae [8]. The daisy or Asteraceae family includes over 1900 genera, providing cooking oils and leaf vegetables, viz., sunflower seeds, artichokes, and lettuce [9]. The genus *Senecio*, meaning “old man” in Latin, is considered one of the largest genera of flowering plants, containing over 1500 species distributed almost worldwide [10]. The *Senecio* species has been used for the treatment of wounds and as vasodilator, anti-inflammatory, and antiemetic preparations in folk medicine [11]. The genus *Senecio* is a rich source of pyrrolizidine alkaloids, cacalolides, and eremophilanolides [12]. *S. petasitis* is native to Mexico and characterized by a yellow daisy-like flower [13]. Despite its therapeutic characteristics, viz., anti-parasitic, anti-inflammatory, anti-cancer, and immunomodulatory [14], *β*-sitosterol glucoside (SG) has received little attention as a natural product due to its low water solubility, which limits its bioavailability; however, this can be enhanced by incorporation into the proposed self-emulsifying delivery system. To the best of our knowledge, there were no studies reported the effect of SG on glucagon levels or insulin resistance in diabetic conditions [15].

Self-emulsifying drug delivery systems (SEDDS) is one of the nanotechnology-based platforms investigated to overcome the problem of poor bioavailability of aqueous insoluble active ingredients. This nanosystem is an isotropic mixture classified as a lipid-based one that is composed of the active agent, the oil, and the surfactant/co-surfactant. SEDDS forms a nanoemulsion upon dilution with the gastrointestinal tract fluids and hence can augment the drug absorption, bioavailability, and performance [16]. In addition, the surfactant used as an ingredient of the formula can interfere with the membranes lipid bilayer and enhance drug permeability through the biological membrane, along with decreasing the possible toxic effects encountered when using the surfactant in the free form [17]. Hence, besides offering a better drug bioavailability, a SEDDS provides different advantages comprising the ease of preparation and possibility of scaling-up without the need for complex procedures or high external energy [18].

The current study evaluated the impact of SG-loaded SEEDS on glucose homeostasis and insulin regulation in order to better understand the role of SG in diabetes management. For the first time, after treatment with SG-loaded nanosystem, we employed insulin tolerance tests (ITTs) to measure insulin sensitivity and the homeostatic model assessment (HOMA) to describe insulin resistance.

## 2. Materials and Methods

### 2.1. Plant Material

*S. petasitis* aerial parts were collected from El-Orman botanical garden in Cairo (Egypt). The plant was kindly identified by Prof. Dr. M. El-Gebally, a botanist specialist, and confirmed with published literature [10]. A voucher sample (SP-09-07-13-05) was kept in the Herbarium of El-Orman botanical garden, Cairo, Egypt.

### 2.2. Chemicals

Streptozotocin (STZ) (>98%), linoleic acid (LA), propylene glycol (PG), and Tween 80 (T80) were purchased from Sigma-Aldrich (St. Louis, MO, USA), and insulin (Actarapid) was purchased from a pharmacy. Acconon^®^ MC8-2 EP/NF (polyoxyethylene-8 caprylic/capric glycerides) and Captex 200^®^ (propylene glycol dicaprylocaprate) were kindly offered by ABITEC Corporations, Cleveland, OH, USA, while Labrafil^®^ M1944LS (decyl polyglucoside) was a free sample from Gattefosse (Saint-Priest, France). All other chemicals used were of analytical grade.

### 2.3. Experimental Animals

Forty male Wistar albino rats, weighing 150–170 g, were obtained from the National Research Centre’s animal house. Animal handling followed the Medical Ethical Committee of Egypt’s National Research Centre’s ethical guidelines. The animals were housed separately and given free access to food and water. The protocol of experimental conditions was accepted by the National Regulations on Animal Welfare and Institutional Animal Ethical Committee (IAEC) (Approval no: 6443042021).

### 2.4. Extraction and Isolation

The lyophilized pulverized aerial parts of *S. petasitis* (800 g) were extracted by ethanol (2 × 5 L), then the concentrated extract (90 g) was suspended in distilled water and fractionated using methylene chloride (3 × 400 mL). Column chromatography packed with silica gel (500 g, 70–230 mesh, 4 × 100 cm) was used to isolate the SG from methylene chloride fraction (30 g) through gradient elution of methylene chloride and increasing percentage of methanol. The eluted fractions were monitored using thin layer chromatography (TLC, 250 μm thickness, KGF Silica gel 60, Merck, Darmstadt, Germany) sprayed with 10% H_2_SO_4_. The fraction eluted with 80% methylene chloride yielded SG (95.6% purity) as white crystals precipitated by methanol.

### 2.5. Spectroscopic Characterization

Spectroscopic techniques such as infrared (IR), and proton nuclear magnetic resonance (^1^H-NMR and ^13^C-NMR) were employed to explore the structure of the SG. IR spectrum was acquired on a Shimadzu IR-400 spectrometer (Kyoto, Japan), and the ^1^H-NMR and ^13^C-NMR spectra were recorded in DMSO-d_6_ on Bruker Ascend TM400/R (Rheinstetten, Germany) at 400 MHz and 100 MHz, respectively.

### 2.6. Preparation of SEDDS

The surfactant, co-surfactant, and oil combination (S/CoS/oil) with a ratio of 45:45:10 (*w*/*w*/*w*) was used to prepare the SEDDSs. Tween 80 (T80) and linoleic acid (LA) were used as the surfactant and oil, respectively, while propylene glycol (PG), Acconon^®^ MC8-2 EP/NF (AC), Captex ^®^200 (C200), and Labrafil^®^ M1944LS (LB) were used as the co-surfactants. The ternary mixtures were stirred by vortex mixing (Julabo Labortechnik, Seelbach, Germany) and left 24 h for equilibrium at room temperature to form homogenous isotropic mixtures [19].

### 2.7. Infinite Dilution Capacity

The infinite dilution capacity of the prepared systems was assessed based on the previously documented visual method [20]. Each tested system (1 g) was diluted gradually with 20 mL of distilled water at 37 ± 0.5 °C with continuous stirring using a magnetic stirrer. Only systems forming a clear nanoemulsion within 1 min were selected to continue the evaluation.

### 2.8. Percentage Transmittance

The percentage transmittance was measured at 560 nm using a Shimadzu UV spectrophotometer (2401/PC, Tokyo, Japan) and deionized water was used as a blank. The selected SEDDS formulation (1 mL) was diluted with distilled water (1:100).

### 2.9. Drug Loading

The drug-loaded SEDDS was prepared by adding SG to the selected system (10 mg/mL) with continuous stirring using a magnetic stirrer at 40 °C until the formation of a clear and transparent system.

### 2.10. Droplet Size Determination, Polydispersity Index, and Zeta Potential

The mean droplet size and the size distribution expressed as polydispersity index (PDI), in addition to the zeta potential (Z) of the selected system were measured using a Zetasizer (Nano-ZS, Malvern Instruments Ltd., Malvern, UK) after doing the suitable dilution with distilled water (1:10).

### 2.11. In Vitro Release Studies

Release studies were done by the dialysis bag diffusion method widely used to test drug release from nanosystems [21,22]. The cellulose membrane (12,000–14,000 MWT) was filled with 1 mL of the drug-loaded SEDDS or the drug suspension containing an equivalent amount of the drug, then both ends of the bags were tied, and the dialysis bags were placed in beakers containing 100 mL phosphate buffer (pH 6.8)/Tween 80 (2.5% *w*/*v*) [23] to assure sink conditions (rotation at 100 rpm for 24 h at 37.0 ± 0.1 °C). At different time intervals up to 24 h, samples (2 mL) were drawn and replaced with an equal volume of fresh medium. A similar set was run in parallel for the unmedicated SEDDS (blank) following the same experimental conditions to eliminate any possible interference. The cumulative amount of drug released was quantitatively determined at 266 nm using Shimadzu UV-2401PC and release profiles were plotted. The experiments were carried out in triplicate and data was expressed as mean value ± S.D.

### 2.12. Transmission Electron Microscopy (TEM)

The micro-morphological structure of the selected nanosystem was investigated by transmission electron microscopy (JEM-1 230; Jeol, Tokyo, Japan). A droplet of diluted preparation was placed on carbon film-coated copper grids and negatively stained with phosphotungstic acid solution 2% (*w*/*w*), and then allowed to dry before examination.

### 2.13. Induction of Diabetes

After acclimatization for a week, 40 rats were divided equally into five groups (8 rats for each) as follows: normal control (G1), streptozotocin (STZ)-induced diabetic control (G2), SG suspension-treated diabetic (20 mg/kg) (G3) [24], unmedicated nanosystem-treated diabetic group (G4), and medicated nanosystem-treated diabetic (G5).

The animals in the normal control group were fed normal chow pellets for the duration of the experiment. The remaining animals, on the other hand, were fed a high-fat diet (HFD) for two weeks to induce insulin resistance. The compositions of HFD were as follows: sucrose 20%, cholesterol 2.5%, lard fat 10%, and powdered normal chow pellets 67.5% [25], and 25% fructose in drinking water [26]. The diet was kept up until the end of the experiment. T2DM was next produced in the rats by giving them 45 mg/kg body weight (BW) of STZ (Sigma-Aldrich, St. Louis, MO, USA) [27]. STZ was freshly dissolved in 0.1 mol/L sodium citrate buffer for this experiment (pH 4.5). Rats with blood glucose levels above 250 mg/dL seven days after induction of diabetes were regarded to have T2DM. Once diabetes was proven, all treated groups (6 rats for each) were administered by gavage needle for 21 days.

### 2.14. Collection of Blood and Tissue Samples

At the end of the experimental period, blood samples were collected from the eyes of all rats. Blood was drawn through cardiac puncture and immediately stored on ice for 3 h before being centrifuged for 15 min at 3000 rpm. The serum was then isolated and kept at –80 °C until further examination. For histopathological analysis, a small sample of pancreatic tissue from each animal was cut and stored in a 10% neutral buffered formalin solution at room temperature. Throughout the preservation period, each pancreas tissue sample’s neutral buffered formalin was replenished weekly.

### 2.15. Measurements of Biochemical Parameters

After 12-h fast, fresh blood samples were taken from the rats’ tail veins. Plasma glucose levels were measured using a blood glucose meter. In serum rats, insulin levels were determined using an enzyme-linked immunosorbent assay (ELISA) with a rat insulin ELISA kit (ELR-Insulin, RayBio^®^, Norcross, GA, USA) and glucagon levels were determined using an enzyme-linked immunosorbent assay (ELISA) with a rat glucagon ELISA kit (E-EL-R0523, Elabscience^®^, Bethesda, MD, USA). The insulin sensitivity of diabetic rats was measured using the homeostasis model assessment-insulin resistance (HOMA-IR) equation [28]:

(Fasting plasma insulin (μIU/mL) × Fasting blood glucose (mg/dL))/405.

In the ITT experiment, after fasting for 8 h, the rats were intraperitoneally injected with insulin at 0.5 IU/kg body weight [29], and blood samples were obtained at 0, 15, 30, and 60 min to evaluate glucose levels.

### 2.16. Determination of Serum Malondialdehyde (MDA)

Lipid peroxidation levels were determined in serum colorimetrically by measuring the content of thiobarbituric acid reactive product, MDA at 534 nm according to the method of Ohkawa, et al. [30] by using MDA kit (Cat no MD 25 29, Biodiagnostic, Dokki, Giza, Egypt).

### 2.17. Determination of Serum Catalase

Serum Catalase levels were determined colorimetrically according to the method of Aebi [31] by using a catalase kit (Cat no CA 25 17, Biodiagnostic, Dokki, Giza, Egypt).

### 2.18. Histopathological Examination of Pancreatic Tissue

Autopsy samples were obtained from the pancreas of rats in various groups and preserved for 24 h in 10% formal saline. After washing with tap water, dehydration was accomplished using serial dilutions of alcohol (methyl, ethyl, and absolute ethyl). Specimens were rinsed in xylene and embedded in paraffin for 24 h at 56 °C in a hot air oven. By using a sledge microtome, paraffin bee wax tissue blocks were formed for sectioning at a thickness of 4 microns. The tissue sections were mounted on glass slides, deparaffinized, and stained with hematoxylin and eosin for examination under a light electric microscope [32].

### 2.19. Statistical Analysis

Using software program GraphPad Prism (version 8.0), the data was analyzed by one-way analysis of variance (ANOVA) followed by Tukey comparison test. The difference was considered significant when *p* < 0.05.

## 3. Results and Discussion

### 3.1. Structure Identification of β-Sitosterol Glucoside

The total extract of *S. petasitis* (F: Asteraceae) aerial parts was chromatography fractionated and then purified, resulting in white needle crystals. IR spectrum revealed bands at 3384 and 1719 cm^−1^ owing to hydroxyl and ester groups, respectively. ^1^H-NMR (400 MHz, DMSO-d_6_) spectrum (Figure S1) exhibited six methyl (CH_3_) signals, viz., two singlets at *δ* 0.63 and 0.94, three doublets at *δ* 0.80 (*J* = 6.2 Hz), 0.84 (*J* = 6.1 Hz), and 0.87 (*J* = 3.1 Hz), in addition to one triplicate at *δ* 0.91 (*J* = 6.8 Hz). Moreover, singlet olefinic proton at *δ* 5.31 and doublet anomeric proton at *δ* 4.24 (*J* = 7.6 Hz) were detected. Regarding the ^13^C-NMR (100 MHz, DMSO-d_6_) spectrum, six methyl (CH_3_) signals were noticed at *δ* 11.82, 19.02, 19.18, 12.03, 18.69, and 19.75. Olefinic and anomeric carbons exhibited signals at *δ* 121.25 and 100.81, respectively. The aforementioned spectroscopic data was in accordance with previously published findings [33,34], indicating the identity of the compound as *β*-sitosterol glucoside.

### 3.2. Assessment of Self-Emulsification

The selection of convenient components to prepare the SEDDS is necessary to obtain a safe and effective nanosystem for pharmaceutical use. The systems under investigation were prepared using the oil and the surfactant in combination with different co-surfactants. The comparison between them was done based on the self-emulsifying capacity of the systems. The successful SEDDS should form a monophasic clear liquid upon dilution with water to allow for the active ingredient to be in a solubilized form upon contact with the gastrointestinal fluid after oral intake. Previous studies proved the crucial role of the co-surfactant, as it can maintain a stable uni-phase oil/water emulsion and form a flexible dynamic layer by diminishing the interfacial tension of the surfactant, so the drug incorporated in this high-energy nanosystem can easily diffuse through the flexible film formed between the phases, which facilitates its partitioning [35]. In addition, it can reduce the required number of surfactants and hence decrease their possible harmful effect.

It was reported that in a conventional emulsion, the different phases tend to separate upon dilution to reduce the interfacial area and the system’s free energy. Controversially, in case of the SEDDS, the emulsification process can occur spontaneously, as the free energy needed to form the nanoemulsion is low [36].

Based on the visual examination to test the self-emulsification process upon dilution, it was detected that among all the prepared systems, only the one prepared with PG as a co-surfactant formed a clear nanoemulsion. The higher hydrophilicity of PG (short chain) compared to the other tested co-surfactants can explain its greater success. It was proved that a surfactant/co-surfactant mixture with higher hydrophilicity or hydrophilic lipophilic balance (HLB value) will help the formation of O/W type nanoemulsions [37]. The use of PG as a co-surfactant has previously proved a success in the preparation of this type of nanocarrier [38,39]. In another study, it was indicated that PG was essential to promote microemulsion formation as it increased the interfacial fluidity of the surfactant. Because of its high hydrophilicity, PG molecules are expected to partition into the aqueous phase and can also be partially incorporated into the polar parts of the surfactant layer, which reduces the rigidity of the condensed interfacial film [40]. Hence, this SEDDS was selected to continue the study.

### 3.3. Percentage Transmittance

The percentage transmittance of the selected SEDDS after dilution with distilled water (1:100) was 99.28 ± 0.20%. Such a value can prove the formation of a clear nanoemulsion and indicate the successful preparation of a self-emulsifying drug delivery system.

### 3.4. Droplet Size Determination

**Figure 1:**
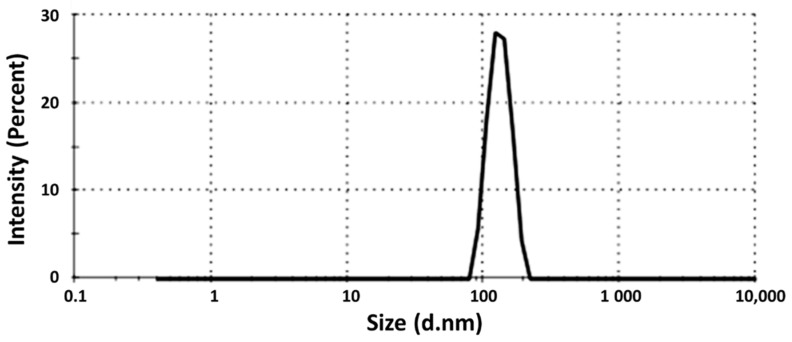
Particle size of the selected drug-loaded SEDDS.

The mean droplet size of the selected SG-loaded SEDDS was found to be 134 ± 15.2 nm, as shown in Figure 1. The nanoemulsion droplet size is a crucial factor influencing the self-emulsification process, and a small size can enhance the stability and facilitate the emulsification of the nanosystem [19,41].

**Figure 2:**
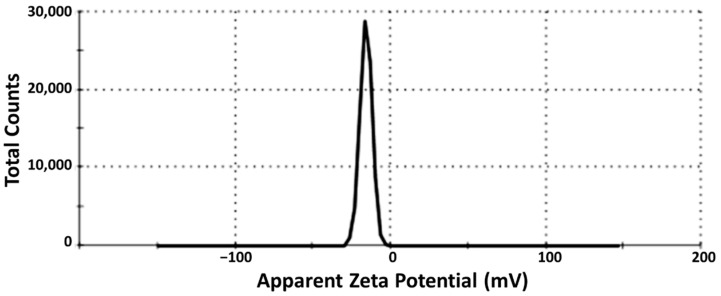
Zeta potential value of the selected drug-loaded SEDDS.

The PDI was found to be equal to 0.296 ± 0.02, such a small value (≤0.3) showing the monomodal size of the tested nanosystem which indicates a homogenous uniform distribution. The Zeta potential (Z) value was −18.8 ± 3.80 mV (Figure 2) which can indicate the physical stability of the tested drug-loaded SEDDS and a low tendency to aggregate.

### 3.5. In Vitro Release Study

**Figure 3:**
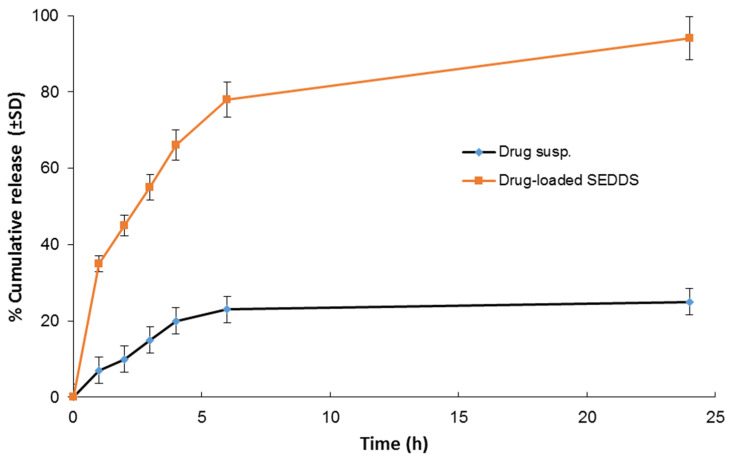
In vitro release study of the drug from the prepared SEDDS compared to the drug suspension.

As seen in Figure 3, the prepared SG-loaded SEDDS significantly enhanced the drug release (~4-fold). After 24 h, the mean cumulative drug release was almost complete compared to only 25% in case of the free drug suspension, which can be explained by the capability of self-emulsifying drug delivery systems to solubilize aqueous insoluble drugs with the formation of fine-droplets nanoemulsions [19,42]. This can enhance drug absorption and bioavailability after oral administration and, in turn, ameliorate the biological efficacy.

### 3.6. Transmission Electron Microscopy

**Figure 4:**
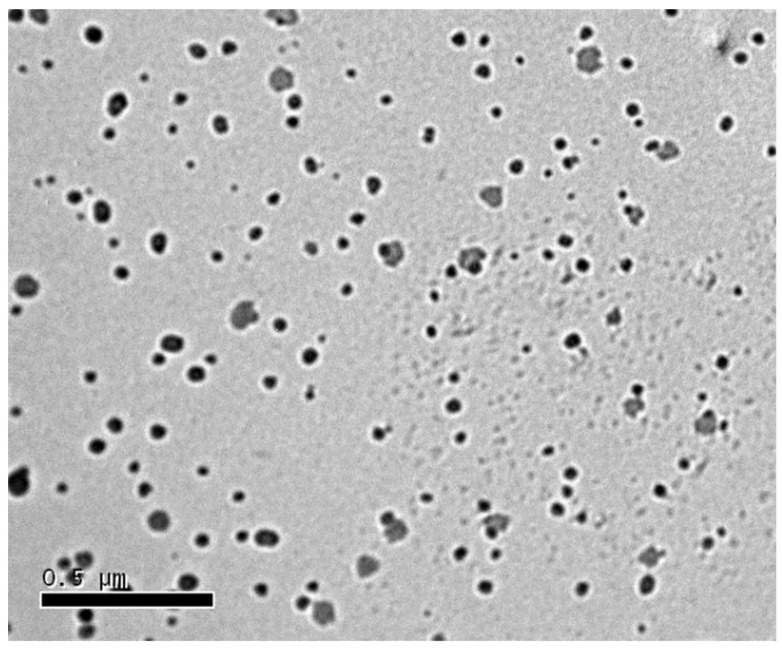
Transmission electron microscopy (TEM) photograph of the tested drug-loaded SEDDS.

Figure 4 illustrates the microphotograph of the tested drug-loaded SEDDS. Almost spherical and separated dark spots, with a size approaching that was recorded by the Zetasizer, can be seen.

All the displayed tests confirm the nanoencapsulation of the drug. In vivo studies comprising antidiabetic effect in streptozotocin-induced diabetic rats were performed to compare the efficacy of the drug-loaded SEDDS to the free drug suspension.

### 3.7. Effects of β-Sitosterol Glucoside on Body Weight and Serum Glucose

**Figure 5:**
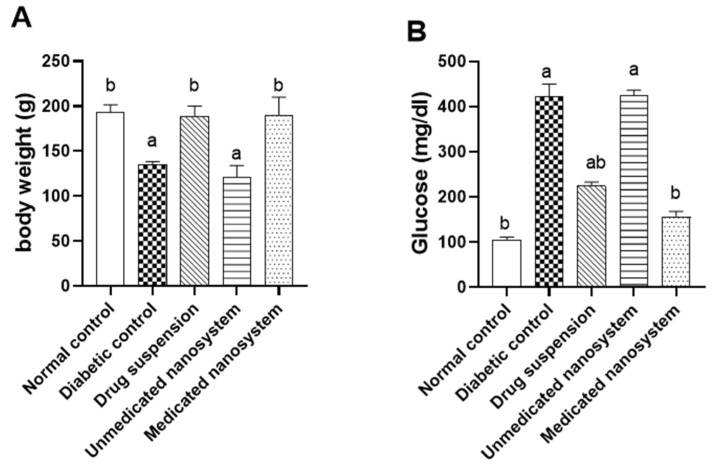
Effect of *β*-sitosterol glucoside on body weight (**A**) and blood glucose level (**B**) in STZ-induced diabetic rats. Each value denotes mean ± SEM (*n* = 6). Statistical analysis was carried out by one-way ANOVA followed by Tukey’s post-hoc test. ^a^ significantly different from normal control. ^b^ significantly different from diabetic control at *p* < 0.05.

The effects of SG on body weight and serum glucose in STZ-induced diabetic rats were measured at the end of the experiment (Figure 5). There was a significant decrease (*p* < 0.05) in the body weight in the diabetic group with a reduction of 29.9% compared to the normal control group. On the other hand, the diabetic group treated with medicated nanosystem and drug suspension showed a significant increase (*p* < 0.05) in its weight (40.4% and 39.5%, respectively) compared to the diabetic control group (Figure 5A). Alternatively, there was a significant increase (*p* < 0.05) in the blood glucose level in the diabetic group compared to the control group and the diabetic group was ~4-fold higher than the normal control group. This level was significantly decreased (*p* < 0.05) in the diabetic group treated with medicated nanosystem and drug suspension when compared to the diabetic control group with a reduction of 63.2% and 47.1%, respectively (Figure 5B). The effect of medicated nanosystem on blood glucose was more comparable to drug suspension.

In our study, we observed that streptozotocin-induced diabetes results in decreased body weight, which is consistent with Luo, Yang, Tang, Yao, Kong, He and Zhou [25], Abdelmageed, et al. [43]. This weight loss might be due to changes in body mass of T2DM rats [44]. Interestingly, treatment of diabetic rats with medicated nanosystem and drug suspension improved body weight when compared with untreated diabetic rats.

T2DM is a complex metabolic disorder that is characterized by high blood glucose, mainly as a result of the dysfunctional secretion and action of insulin. To avoid complications, the major diabetes therapeutic interventions are to maintain blood glucose levels, improve *β*-cell function, and minimize insulin resistance. Meanwhile, drugs used to treat diabetic diseases must enhance glucose homeostasis and insulin sensitivity in addition to lowering blood glucose levels [45].

### 3.8. Effect of β-Sitosterol Glucoside on Serum Insulin, HOMA-IR, and Insulin Tolerance

**Figure 6:**
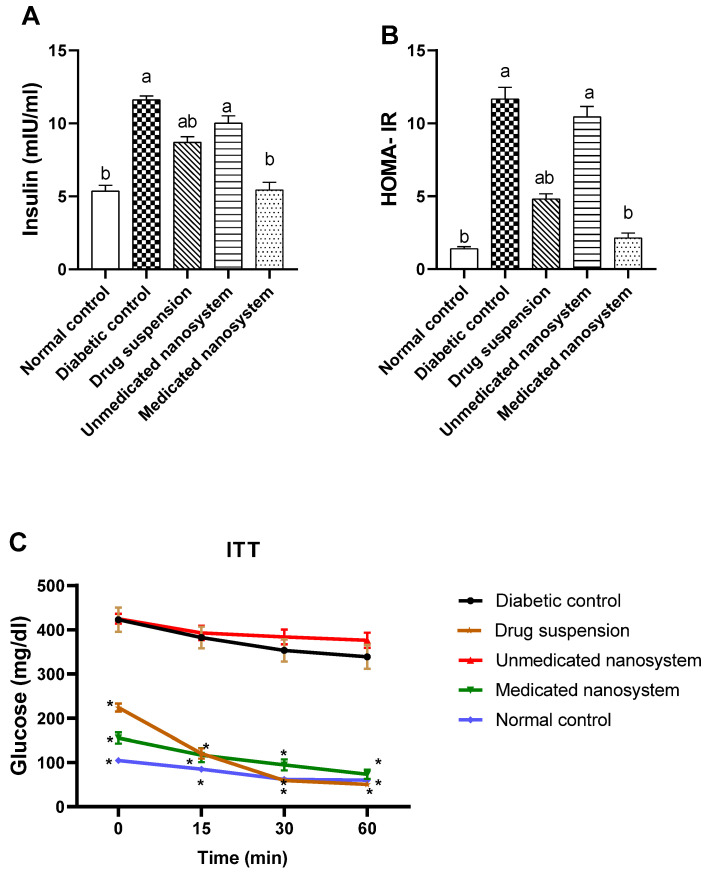
Effect of *β*-sitosterol glucoside on serum insulin (**A**), HOMA- IR (**B**), and ITT (**C**) in STZ-induced diabetic rats. Each value denotes mean ± SEM (*n* = 6). Statistical analysis was carried out by one-way ANOVA followed by Tukey’s post-hoc test. ^a^ significantly different from normal control. ^b^ significantly different from diabetic control at *p* < 0.05. * significantly different from diabetic control at *p* < 0.05 for ITT.

The level of insulin was significantly increased (*p* < 0.05) in the diabetic group by about 2.16-fold (Figure 6A) compared to the normal control group. However, the medicated nanosystem group and drug suspension showed a lower level of insulin content than the diabetic control group with a reduction of 53.11% and 25.13%, respectively. Regarding HOMA-IR (Figure 6B), there was a significant elevation (8.3 fold higher) in the diabetic group compared to the normal control group (*p* < 0.05), indicating induction of insulin resistance. Upon treatment with medicated nanosystem and drug suspension, there was a significant reduction in HOMA-IR with a percentage decline of 81.60% and 58.75%, respectively, compared with the diabetic control group. However, the effect of medicated nanosystem on insulin resistance showed a more prominent effect.

In ITT, insulin administration (0.5 IU/kg, i.p.) to normal (G1), diabetic (G2), and treated animals (G3–G5) produced a decrease in blood glucose levels (Figure 6C), although the rate of decline in blood glucose was significantly higher in normal rats (*p* < 0.05, *n* = 6) compared to the diabetic untreated rats (Figure 6C). The blood glucose level in the medicated nanosystem group and drug suspension group were significantly lower (*p* < 0.05, *n* = 6) after insulin loading than the level in the diabetic rats at the 15-, 30-, and 60-min time points. These results indicate that medicated nanosystem and drug suspension enhanced insulin sensitivity and glucose utilization significantly (*p* < 0.05) over the complete period during the ITT compared to diabetic control rats.

In comparison to the control group (G1), the diabetic control group (G2) had significantly higher fasting glucose and insulin levels, as well as higher HOMA-IR values. T2DM is marked by a progressive loss of insulin action, referred to as insulin resistance, as well as compensatory hyperinsulinemia and fasting hyperglycemia [46]. The high HOMA-IR measurement reflects these two metabolic abnormalities. When compared to the diabetic control group, medicated nanosystem treatment group (G5) and drug suspension group (G3) lowered fasting hyperglycemia and basal insulin levels, as well as HOMA-IR values, implying its capacity to alleviate insulin resistance and restore *β*-cell secretory function. Medicated nanosystem group restored both insulin levels and HOMA-IR values to near normal and have the best effect. Moreover, medicated nanosystem treatment enhanced insulin sensitivity and promoted glucose utilization in T2DM rats, as shown by significant decreases in glucose levels in 15-, 30-, and 60-min time points in the ITT test. ITT evaluates insulin resistance and could be a useful tool for predicting the efficacy of insulin sensitizers [47].

### 3.9. Effects of β-Sitosterol Glucoside on Serum Malondialdehyde and Catalase Levels

**Figure 7:**
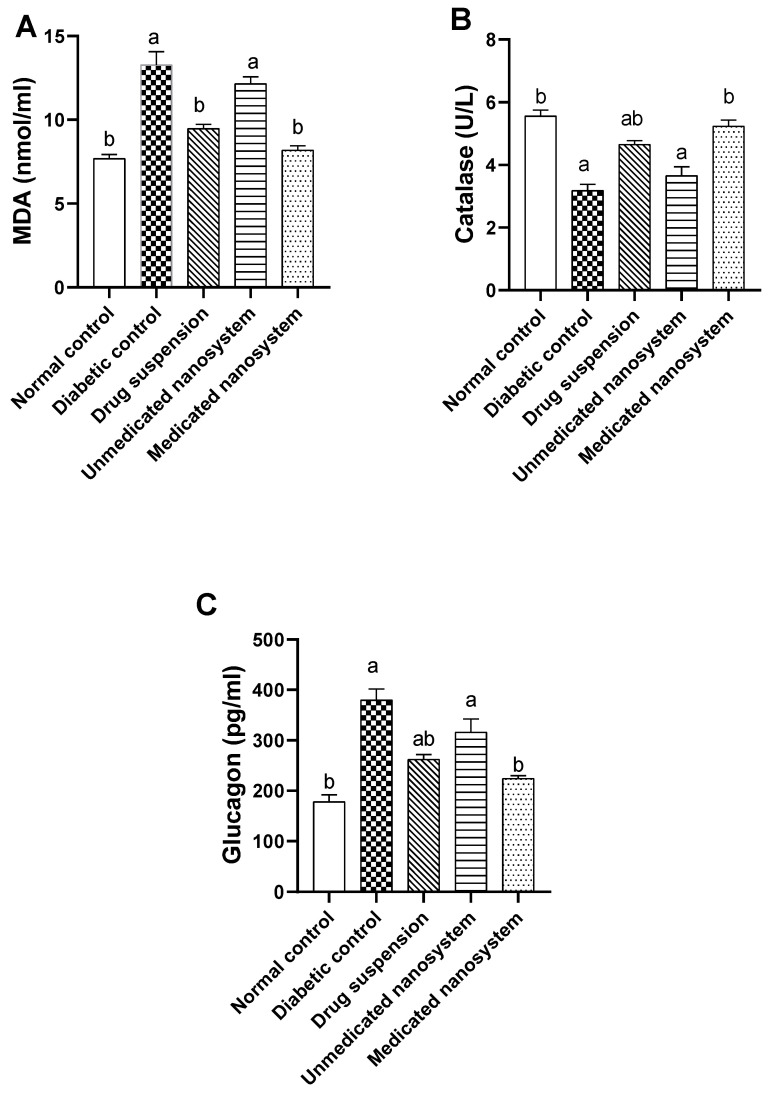
Effect of *β*-sitosterol glucoside on MDA (**A**), catalase (**B**), and glucagon levels (**C**) in STZ-induced diabetic rats. Each value denotes mean ± SEM (*n* = 6). Statistical analysis was carried out by one-way ANOVA followed by Tukey’s post-hoc test. ^a^ significantly different from normal control. ^b^ significantly different from diabetic control at *p* < 0.05.

Oxidative stress was assessed by measuring serum malondialdehyde and catalase levels. Compared with the normal control group, serum MDA content of the diabetic group was markedly elevated (1.7-fold) (*p* < 0.05) (Figure 7A), while serum catalase (Figure 7B) was significantly (*p* < 0.05) lower (0.57-fold) than those of normal control group. Medicated nanosystem and drug suspension treatment significantly suppressed lipid peroxidation levels (38.31% and 28.59%, respectively) in comparison with the diabetic untreated group. On the other hand, treatment with medicated nanosystem and drug suspension caused a significant increase in serum catalase levels (64.45% and 46.21%, respectively) compared to the diabetic control group. Oral administration of medicated nanosystem significantly (*p* < 0.05) restored these levels to near-normal values.

Insulin resistance, increasing *β*-cell dysfunction, poor glucose consumption, and T2DM have all been linked to oxidative stress [48]. Chronic hyperglycemia boosts tissue ROS and lipid peroxidation, impairing insulin action [49]. Moreover, antioxidant enzymes such as CAT play a crucial role in overcoming oxidative stress [50]. In the present study, as shown in Figure 7, in comparison to the normal control group, the serum of the diabetic control group exhibited a large increase in MDA, a lipid peroxidation product [51], and a significant decrease in catalase activity. These findings are supported by previous reports [52,53,54]. The medicated nanosystem-treated group mitigated oxidative stress in the serum of T2DM rats by decreasing the serum level of MDA and subsequently increasing the serum level of CAT.

### 3.10. Effects of β-Sitosterol Glucoside on Serum Glucagon Levels

Further, the glucagon activity was investigated in the serum of tested groups (Figure 7C). Compared with normal control rats, glucagon level was significantly increased (*p* < 0.05, 2.13-fold) in diabetic untreated rats. On the other hand, glucagon levels were significantly decreased in diabetic rats treated with drug suspension (30.91%) and medicated nanosystem (40.85%) compared to the diabetic control group.

Further, to understand the mechanism of action of the SG, we measured the glucagon levels. The elevation of the activity of the glucagon in diabetic rats was reported in our study, and this finding is consistent with previous studies [55,56]. The upregulation of glucagon secretion may contribute to problems with glucose homeostasis [57].

Furthermore, increasing blood glucagon concentrations are linked to enhanced insulin resistance and raised glucose concentrations in both normal and T2DM participants [58], suggesting that glucagon may play a role in T2DM pathogenesis. In this study, medicated nanosystem administration reduced glucagon levels, implying that SG can alleviate insulin resistance, which is a complication of diabetes.

### 3.11. Effects of β-Sitosterol Glucoside on Pancreatic Histological Changes

**Figure 8:**
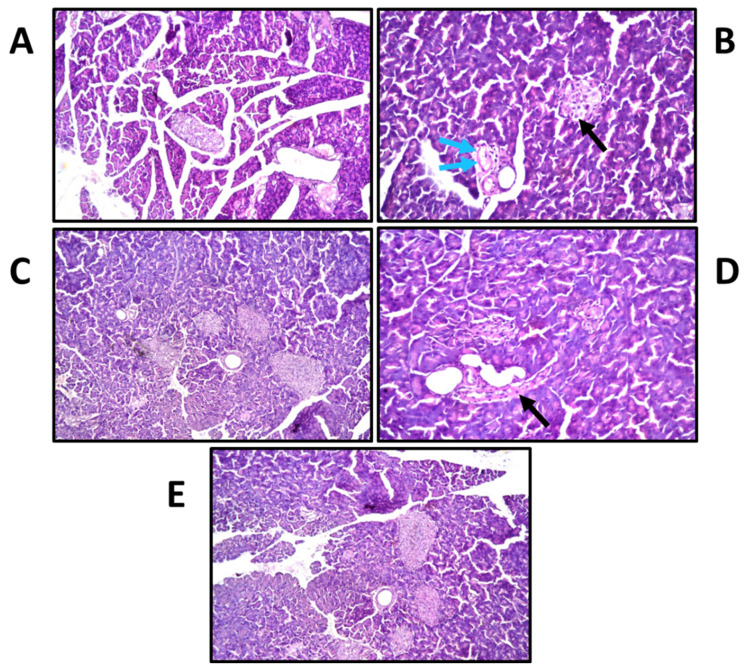
Histopathology of rat pancreatic tissue stained with hematoxylin and eosin determined by light electric microscopy (×40); (**A**): normal control revealing normal histological structure of cells; (**B**): diabetic control demonstrated atrophy (Black arrows), oedema (Blue arrows), and a drastic decrease in the number of the islets of Langerhans cells; (**C**): diabetic + drug suspension exhibited a remarkable recovery; (**D**): diabetic + unmedicated nanosystem resulted in atrophy (Black arrows) and regression in the size contour of the islets of Langerhans cells; and (**E**): diabetic + medicated nanosystem, revealing no histopathological alteration in the islets of Langerhans cells compared to normal control.

Histological changes were screened to support results of the biochemical results. The photographs of histology sections are depicted in Figure 8. Histopathological examination revealed that the control group had a normal histological structure of the acini and duct system (Figure 8A). On the other hand, the diabetic group demonstrated atrophy and regression in the number of the islets of Langerhans cells with the surrounding intact acini (Figure 8B), in association with focal inflammatory cells infiltration and oedema surrounding the dilated blood vessels in the stroma between the lobules. SG suspension exhibited a remarkable recovery compared to diabetic untreated rats, with a mild degree of inflammation (Figure 8C). Treatment with blank (non-medicated nanosystem) resulted in atrophy and regression in the size contour of the islets of Langerhans cells (Figure 8D). Focal inflammatory cells infiltration was replaced by the regressed atrophied islet of Langerhans in the lobules. The interlobular stromal tissue showed focal oedema with inflammatory cells infiltration. Severe dilatation and congestion were detected in the stromal blood vessels associated with perivascular hemorrhages, oedema, and inflammatory cells infiltration. On the other hand, the medicated nanosystem (Figure 8E) has protected the tissue damage and there was no histopathological alteration in the islets of Langerhans cells and surrounding acini.

The protective effect of SG on cells of the islets of Langerhans is also confirmed by histopathology of pancreatic cells. The STZ-induced diabetic rats had atrophy and regression in the number of the islets of Langerhans cells, whereas rats given medicated nanosystem showed no alteration in islet growth and significantly reduced pancreatic *β*-cell damage.

In plants, phytosterols are essential metabolites reported as bioactive compounds against various pathogens and diseases [59]. Additionally, phytosterols are present in the human diet, and researchers have explored their absorption and metabolism [60,61]. It is recognized that SG is safe when taken orally, albeit with low bioavailability [62]; its incorporation into a self-nanoemulsifying delivery system enhanced the solubility and biological effect. Furthermore, our in vitro release findings indicate improved delivery of SG through increased dissolution and absorption. In vitro release study is a potent and valuable tool for monitoring clinical performance as a surrogate for in vivo pharmacokinetics [63]. Thus, a good linear relationship was observed upon investigating the in vitro/in vivo correlations [64,65].

Increasing oxidative stress aggravates diabetes and its complications. Therefore, antioxidants are commonly used as supplementary medicine especially in chronic diabetes [66]. SG was reported as an antioxidant inducing various therapeutic properties, viz., neuroprotective, analgesic, anti-inflammatory, and chemopreventive activities [14]. However, no studies reported any adverse effects of SG [67].

The current study focused on the efficacy of SG-loaded SEEDS on insulin resistance and glucagon levels for the first time in treating diabetes. Thus, experiments were designed to investigate these effects of SG in HFD-fed diabetic rats because HFD feeding causes insulin resistance and can be used to establish T2DM animal models [68]. A myriad of pathways and mechanisms can be considered as underlying reasons for enhanced insulin sensitivity and glucose homeostasis. For instance, insulin stimulates the translocation of GLUT4 (Glucose transporter type 4), which elevates glucose uptake in skeletal muscles. Thus, defective GLUT4 translocation exacerbates insulin resistance [69]. SG was reported to induce an estrogen like effect and increase GLUT4 translocation through PI-3K/AKT signaling in skeletal muscles [70]. In insulin resistance and obesity, c-Jun *N*-terminal kinase (JNK) is one of the most studied signal transducers [71]. SG was reported to possess an apoptotic effect in cancer cells through activating JNK signaling [72]. Nowadays, pharmacological management of diabetes is performed by inhibiting *α*-glucosidase and *α*-amylase, which aggressively delays the digestion of polysaccharides to absorbable simple sugars [73]. It was recently discovered that SG inhibited *α*-amylase and *α*-glucosidase [74], developing a potential hypoglycemic effect. By inducing inflammatory cytokines, NF-κB (Nuclear factor kappa B) may lower insulin sensitivity [75]. However, a recent study revealed that SG had no effect on the NF-κB signaling pathway [76]. Lastly, another study demonstrated the hypoglycemic effect of SG on improving some hematology parameters, viz., lipid profile, creatinine, and blood urea [77]. Therefore, the proposed mechanisms underlying the SG-induced effect on glucagon levels, insulin tolerance, and glucose homeostasis require further studies for clarification.

## 4. Conclusions

This study provides the first insight into *β*-sitosterol glucoside’s effect on insulin resistance and glucagon levels in diabetes progression. *β*-Sitosterol glucoside extracted from *Senecio petasitis* was loaded in self-nanoemulsifying delivery systems to overcome its low aqueous solubility. The nanosystem showed an enhanced in vitro release of *β*-sitosterol glucoside compared to that of the drug suspension, providing a likely sensible model for pharmacokinetic aspect of *β*-sitosterol glucoside-loaded SEDDS in living organisms. The results from the HOMA-IR and ITTs revealed that *β*-sitosterol glucoside significantly improved insulin sensitivity and glucose utilization in diabetic rats. However, the hypoglycemic effect of *β*-sitosterol glucoside is regulated by a variety of enzymes and mechanisms. Future research work should investigate key markers in glycolysis, glycogen synthesis/degradation, gluconeogenesis, and glucose phosphorylation/dephosphorylation pathways. By being an antioxidant, *β*-sitosterol glucoside may partially attenuate hyperglycemia, though such a hypothesis should be confirmed by excessive further investigations. Additionally, whether *β*-sitosterol glucoside and its aglycone exhibit synergistic antidiabetic activities should be clarified later. The findings of the in vivo studies done using diabetic rats highlight the superior efficacy of the proposed *β*-sitosterol glucoside-loaded nanosystem compared to the free drug suspension in the suppression and control of T2DM by reducing oxidative stress and insulin resistance, indicating that it might be a promising biopharmaceutical candidate. In our histological study, we discovered that *β*-sitosterol glucoside not only reduces hyperglycemia but also attenuates pancreatic inflammation. Thus, it possesses huge potential for countering the deterioration of insulin resistance. Further clinical studies are recommended to unravel various pathways exerted by *β*-sitosterol glucoside, providing a new vision for the treatment of T2DM.

## Data Availability

All of the data is contained within the article and the Supplementary Materials.

## Supplementary Materials

The following supporting information can be downloaded at: https://www.mdpi.com/article/10.3390/antiox11051023/s1, Figure S1: ^1^H-NMR spectrum of the isolated compound (*β*-Sitosterol glucoside) in DMSO-d_6_.
