# Supplementary material for: β-Sitosterol Glucoside-Loaded Nanosystem Ameliorates Insulin Resistance and Oxidative Stress in Streptozotocin-Induced Diabetic Rats
Source: Antioxidants (Basel). 2022 May 22;11(5):1023. doi: 10.3390/antiox11051023 (PMC9137832; doi:10.3390/antiox11051023)
Supplement: Supplementary file 1 [file antioxidants-11-01023-s001.zip › antioxidants-1714270-supplementary.pdf]

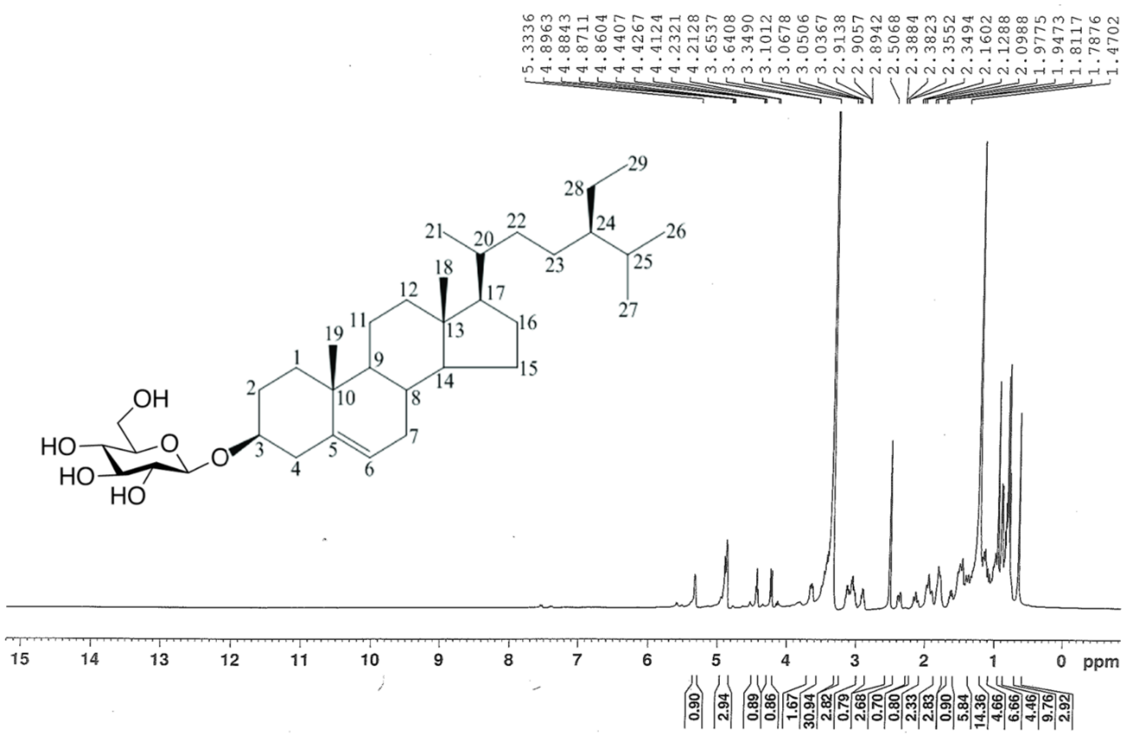

Figure S1. ^{1}H-NMR spectrum of the isolated compound (*β*-Sitosterol glucoside) in DMSO-d_6.
